# Mindfulness cognitive-based therapy combined with metaphor therapy can improve problematic social media use

**DOI:** 10.3389/fpsyt.2025.1503049

**Published:** 2025-02-25

**Authors:** Weirui Xiong, Lu Yu, Jinhui Chai

**Affiliations:** School of Educational Science, Chongqing Normal University, Chongqing, China

**Keywords:** problematic social media use, mindfulness-based cognitive therapy, metaphor therapy, social media, meditation

## Abstract

Problematic Social Media Use (PSMU) refers to an individual’s long-term, high-intensity use of social media. Although this behavior is not pathological, it has a negative impact on the physical and mental health of individuals. In the past, intervention methods used for PSMU often controlled behavior to reduce the usage time of individual social media platforms. However, because psychological needs satisfaction and unreasonable cognition that lead to the problematic use of social media by individuals are not considered, behavior-based treatment models often do not address this problem.

**Method:**

30 participants with PSMU (with another 30 participants as a control group) participated in an intervention program of mindfulness-based cognitive therapy (MBCT) combined with metaphor therapy (MT).

**Results:**

The results showed that these therapies changed the participants’ cognition and behavior and achieved a good intervention effect.

**Conclusion:**

The MBCT combined with MT can be used as an intervention for patients with PSMU.

## 1 Introduction

Social media refers to mobile applications and websites that carry text, images, audio, and videos, allowing users to create, share, and exchange information (1). Popular social media websites include Facebook, Twitter, Instagram, and TikTok. These software programs have an impact on lifestyle and behavioral characteristics. Problematic Social Media Use (PSMU) refers to excessive and uncontrollable use of social media that adversely affects individuals’ lives, work, interpersonal relationships, and mental health. Currently, researchers disagree on whether PSMU is addictive because relationship addiction in Internet addiction is based on social media. Some scholars have suggested that PSMU and Internet addiction are symptoms of addiction (2). Others argue that PSMU does not have a pathological effect on individuals, whereas addiction does (3).

Social media use can alleviate social anxiety to a certain extent (4). However, excessive use of social media and networks can be physically and mentally damaging. This is a popular topic in current research. Hu (5) found that PSMU could physically affect sleep quality. Psychologically, it can cause individuals to experience depression, anxiety, and other negative emotions (6). Song et al. (7) conducted a meta-analysis and found a significant correlation between loneliness and PSMU. Stavropoulos et al. (8) found that individuals with PSMU have lower levels of mental health and self-esteem. Additionally, Kross et al. (9) found that PSMU is associated with maladaptive cognition, increased mental distress, and decreased well-being. Overall, the impact of PSMU on individuals is generally negative. Therefore, social media interventions have become the focus of attention.

To intervene in PSMU, it is important to understand why it occurs in the first place, as this provides a rationale for identifying potential intervention targets. Currently, there are four theories on why PSMU occurs. The first is the compensatory Internet use theory, which holds that when individuals encounter difficulties or obstacles in real life and are unable to fulfill their social and psychological needs, they use the Internet and social media to meet these needs. People who feel lonely or excluded in real life may be more inclined to seek attention and approval through social media (10). According to the theory of compensation, the core intervention for PSMU is to find other ways to meet individuals’ psychological needs.

The second theory is the I-PACE (Individual-Passion, Appraisal, Conation, Environment) model, a framework for understanding and predicting the development of barriers to Internet use. This model argues that the development of Internet use disorders is shaped by interactions among individual characteristics, emotional and cognitive responses to Internet use, and executive functions (11). Individual characteristics include biological psychology, psychopathology, and early childhood experiences. These factors influence how individuals perceive and assess their Internet activities. Internet use triggers emotional and cognitive responses, including impulsivity, cravings, and cognitive biases. When individuals use the Internet to cope with negative emotions or experience satisfaction, these needs may lead them to overuse it. Executive function refers to inhibiting control and decision-making, which are crucial for regulating Internet use. Impairment of these functions leads to losing control over Internet use, making individuals more vulnerable to Internet use disorders. The I-PACE model emphasizes the importance of considering the complex interactions among various factors when understanding and treating Internet use disorders (11).

The third is the use of gratification theory. It emphasizes how individuals actively select social media resources to meet their specific needs and desires and focuses on how social media affects users. This theory emphasizes the initiatives by which individuals actively choose media to meet their needs (12). The fourth theory is the cognitive-behavioral model. The core idea is that cognition (thoughts, beliefs, and attitudes) affects behavior and emotions. Negative or irrational cognition can lead to maladaptive behaviors and negative emotions such as anxiety and depression. By changing these maladaptive cognitive and behavioral patterns, individuals can improve their emotional states, reduce PSMU, and develop healthier social media use habits (13).

Thus, a different understanding of the causes of PSMU leads to different problematic social media interventions. However, intervention studies on PSMU have generally focused on controlling behaviors. For example, the interventions proposed by Stinson and Dallery (14) included encouraging students to reduce their social media use by providing positive feedback or rewards, using smartphone apps to monitor and remind students of their social media use, and encouraging them to find alternative activities to replace the time spent on excessive social media use. In Zhou's (15) study, PSMU was interfered with by banning cell phone use for two hours a day and having to write a diary. Chen et al. (16) used a social-skills training approach. These behavioral control programs have been effective in improving PSMU. However, the main effect of the behavior-based intervention model was to reduce the amount of time individuals spent using social media, which was ineffective in meeting their psychological needs or changing their irrational perceptions. Therefore, other methods should be combined to meet psychological needs or change irrational cognition when controlling for individual behavior. It is necessary to consider both the individual’s behavior and cognition in the intervention method; mindfulness-based cognitive therapy (MBCT) and metaphor therapy (MT) are two kinds of psychotherapy methods that can consider both the behavioral and cognitive aspects.

MBCT is a psychotherapeutic method that combines mindfulness with cognitive-behavioral therapy (CBT). This therapy was initially developed to prevent the relapse of major depressive disorder but has since been used to treat a variety of mental health problems (17). Mindfulness, derived from Buddhism, refers to an individual’s conscious attention to internal and external experiences in the present in a non-judgmental and receptive manner. It encourages individuals to observe their thoughts, feelings, physical sensations, and the environment rather than face these experiences with judgment or criticism (18). MBCT combines the elements of mindfulness and CBT. The first is to develop mindfulness skills that, through meditation and mindfulness practice, help individuals maintain awareness of their thoughts, emotions, and physical feelings in their daily lives. The second is to identify and change automatic thinking modes so that individuals can recognize their automatic negative thinking habits. Third, it helps individuals learn to observe their thoughts in a more objective and nonjudgmental manner. The MBCT emphasizes using specific techniques and strategies to improve individuals’ mental health and quality of life based on their acceptance and understanding of their psychological experiences (19). Liu et al. (20) investigated the effects of loving-kindness meditation on mindfulness, spirituality, and subjective well-being (SWB) of flight attendants. The results showed that loving-kindness meditation positively impacts SWB and spirituality. Moreover, short video app-guided loving-kindness meditation may help improve self-compassion and positive psychological capital and reduce suicidal ideation (21). Chiou et al. (22) and Liu et al. (23) also developed an application to improve depression and enhance spirituality based on the concept of I-Sustainability Design Thinking and the theme of loving-kindness meditation on mindfulness. Chen et al. (24) Chiou et al. (25), and others have conducted similar studies, arriving at the same conclusion. Thus, mindfulness-based intervention methods significantly improve mental health both behaviorally and cognitively. Consequently, in theory, MBCT could intervene in PSMU from both the behavioral and cognitive perspectives. Jones et al. (26) discovered that the awareness component of mindfulness significantly mediated the relationship between each dimension of social media engagement scales—behavioral, emotional, and cognitive—and depression. Therefore, implementing mindfulness-based interventions may be useful in teaching how to engage in social media mindfully and positively. Gómez et al. (27) developed a cooperative serious game to support stress management education and assessed its application among potential users. These findings indicated that learning mindfulness techniques to reduce stress among university students was significant. This study also directly examined the effects of the MBCT on improving PSMU.

In addition, the application of metaphors in psychological counseling and therapy is familiar. Kopp (28, 29) was the first to explicitly use MT and proposed a distinction between visitor- and therapist-generated metaphors. He believes that MT is not a new “therapeutic school”; on the contrary, MT emphasizes the metaphorical communication between the client and the therapist and regards metaphor as a psychotherapeutic perspective and therapeutic intervention central to the changing psychotherapy process (28). Metaphors are often used in acceptance and commitment therapy (ACT). ACT emphasizes the use of metaphors and experiential exercises to help individuals understand pain and treatments in ways that are experiential rather than rational (30). Some researchers consider MT a unique treatment. For example, individual metaphor therapy proposed by Komasi et al. (31) effectively changed people’s irrational beliefs and disastrous thoughts. Xiong (32), in his book *Return to the original state*, specifically addresses the unique role of metaphors in counseling and therapy, arguing that MT is like MBCT and can cause an individual’s behavior and cognition to produce change simultaneously. Therefore, MT can be used as a form of psychotherapy.

In summary, it is not sufficient to intervene in PSMU by controlling for past behavior. This cognitive change occurs at the root of the problem. Therefore, only intervention programs that change both behavioral and cognitive aspects can impact PSMU. This quasi-experimental study examined the effects of MBCT combined with MT on problematic social media.

## 2 Methodology

### 2.1 Participants

An online questionnaire survey examining the prevalence of social media use among college students was conducted with 700 participants. The school’s College Student Psychological Health Center initiated the survey and was approved by the school’s ethics committee. Analysis of the survey results indicated that the average PSMU score of the 700 participants was 3.47 (SD = 0.72). This suggests that PSMU is prevalent among today’s college students (an average score above three indicates such a problem). Subsequently, volunteers with significant PSMU were identified and recruited based on specific criteria: a score exceeding 60 on the “Problematic Social Media Use Scale” and a self-reported average daily social media usage time of more than four hours. These individuals were informed of the purpose, process, and potential benefits of the study, and their willingness to participate in the intervention and experiment was assessed. Sixty participants were recruited for this study. The 60 participants were randomly divided into an intervention group (N = 30) and a control group (N = 30). The staff composition is presented in **Table 1**.

**Table 1:** Basic information about the participants (N = 60).

Demographic variables	Classification	Experimental group	Control group
Sex	Male	17	16
	Female	13	14
Family sources	Town	8	9
	Rural	9	9
	City	13	12
Is an only child	Yes	18	19
	No	12	11

### 2.2 Measurements

This study adopts the “Problematic Social Media Use Scale” developed by Jiang (33). A total of 20 questions were scored using Likert’s five-point scoring method; the participants’ feelings about each situation were scored as “1 = Completely inconsistent,” “2 = Relatively inconsistent,” “3 = Uncertain,” “4 = Relatively consistent,” and “5 = Completely consistent.” Higher scores indicated more negative scores. Cronbach’s α was 0.91. The reliability and validity of the questionnaire are good.

All participants underwent three measurements. The first was taken before the intervention, the second was taken after the last intervention, and the third was a follow-up measurement taken three months after the end of the intervention to examine the continuing effect of the intervention program.

### 2.3 Experimental design

A quasi-experimental design was used. The participants were divided into two groups: intervention and control. During the experimental period, the intervention group received a group intervention once a week for eight weeks, while the control group did not receive any intervention. Given the ethics of the study, participants in the control group were also administered the same intervention protocol at the end of the experiment and after data collection was completed.

### 2.4 Group intervention program

This study used a group intervention method. The group intervention program combined the MBCT and MT. The basic ideas for the solution are shown in **Table 2**.

**Table 2:** Basic ideas of the group intervention program.

	Topic	MBCT	MT
1	Preparation phase	Introduce MBCT familiar with mindfulness	Introduce MT
2	Go beyond automatic navigation	Introduce automatic navigation mindfulness eating Share emotional feelings	Space metaphor
3	Thinking and direct perception	Introduce two approaches to cognition Body scan	Seed metaphor
4	Identify evasive responses	Mindfulness meditation Introduction and analysis of the circumvention response Complete a list of negative thoughts	Sapling metaphor
5	Accept and let go	Mindfulness meditation Foster acceptance and let go Get along with difficulties	Shark metaphor
6	Emotions and thoughts	Body scan Introduce the mental framework difference between thought and fact	Stone metaphor
7	Turn kindness into action	Mindfulness walking Behavior and emotions Control and pleasure activities	Integration
8	Share and end	Mindfulness meditation Changes in thought patterns	Share and end

The first is the preparation phase. The main aim was to introduce the basic principles of the MBCT and MT, allowing subjects to learn the basic methods of mindful breathing and mindfulness. Prior to this, all participants were required to complete the first assessment and clarify the process and rules of the intervention.

The main goal of the second session was to transcend the “autopilot” lifestyle. The participants were first introduced to automatic navigation and a self-controlled lifestyle and were then given mindfulness eating exercises. Introducing an automated navigation lifestyle to the participants can lead to a lack of opportunities for self-reflection and growth, which ignores new possibilities and changes and may even cause unhealthy habits and behavioral patterns to persist. The reason for adopting this approach is that many individuals with PSMU are in negative automated navigation mode. Practicing mindful eating helped the participants recognize and break out of this habitual lifestyle pattern. The specific method involved observing raisins, touching raisins, smelling raisins, and chewing raisins, all performed at a slow pace; participants were asked to refrain from any judgment of the food or their eating habits throughout the process. At this stage, the participants were asked to imagine the first metaphor, the space metaphor, during the sit-in. The space metaphor is a three-step process. The first step was to ask the participants to imagine themselves as stars floating in a space surrounded by many other stars. The stars seemed to move in the desired direction. Only one star wandered aimlessly and did not know where to float. A few minutes later, in the second step, the subjects still imagined themselves as stars floating in space, but this time, they could see a large, beautiful planet in front of them, which was the star’s destination and home. In the third step, the subject does not have to pay attention to the stars around it or to the destination. Participants experienced feelings of being in their homes and destinations. At the end of the process, team members shared their feelings. This metaphor is related to the target, and the purpose of using this metaphorical story is to enable individuals to reflect on their own goals. When people have clear goals, they understand which things in life are related to the goals and which they should spend more time on.

The main goal of the third session was to allow the subjects to understand the two different ways of thinking and direct perception and learn how to do so. The reason for adopting this strategy is that direct perception can help people avoid misunderstandings or biases that may arise from over-reliance on abstract thinking, as it provides a pathway for direct contact with reality; moreover, direct perception is often accompanied by emotional responses, which can add depth and richness to the experience. This can assist individuals immersed in social media in stepping out of the illusory online world and directly confronting the real world. At this stage, the participants needed to learn about body scanning and apply it to their daily lives. Body scanning is a mindfulness meditation exercise that involves closing the eyes and sitting quietly or lying down flat, sequentially focusing attention on various parts of the body from the top of the head to the tips of the toes to feel, observe, acknowledge, and release tension and discomfort in the body while maintaining awareness of breathing. This brings the whole person back to the present state and achieves the effect of physical and mental relaxation. Simultaneously, the second MT metaphor, the seed metaphor, is learned. The participants first imagined themselves as seeds buried in the ground, in the dark. Participants were guided to experience the seeds carefully. After a certain period, the subjects imagined that the seed would finally sprout after a constant struggle, finally seeing sunlight and hearing sound. We then experience the feelings of this seed. Finally, participants imagined themselves as simple seeds, whether buried in the soil or germinated, which reflected the nature of all complete seeds. This metaphor is related to setbacks, and the purpose of using it was to help participants correctly recognize and directly confront, rather than avoid, the setbacks they encounter in their lives. Many PSMUsers turn to the online world to escape setbacks in their lives.

The fourth attempt was to identify avoidance responses. The avoidance response refers to the automatic behavioral reactions of avoidance or escape that an individual adopts when faced with unpleasant or threatening stimuli; doing so is an instinctive self-protection mechanism. Many individuals become immersed in social media because they have encountered unpleasant events in real life, leading to an automatic escape response. To introduce the participants to the avoidance response, an analysis of the avoidance response helps to establish a positive attitude. The participants were guided to engage in mindfulness meditation, fill in a negative-thinking list, and look for their own evasive characteristics. Mindfulness meditation typically involves finding a quiet environment to sit in, maintaining an upright posture, focusing attention on the breath, and feeling the airflow with each inhalation and exhalation while accepting and observing various sensations, emotions, and thoughts in the body without any judgment or reaction. Additionally, at this stage, the participants were guided to learn the sapling metaphor of MT. In this exercise, participants were initially instructed to imagine themselves as young saplings surrounded by large trees, with soft sunlight washing the ground, although the surrounding trees almost completely block the sun. Later, heavy rain and strong winds occurred; however, the participants found that the surrounding trees also blocked heavy rain and strong winds. Finally, the participants imagined they were unique saplings regardless of whether a large tree was nearby. They grow according to their nature and become trees. This metaphor is related to parent-child relationships. The reason for using this metaphor is that many unpleasant events that individuals encounter in real life are associated with parent-child relationships. This metaphor can help participants gain a more objective and accurate understanding of parent-child relationships.

The goal of the fifth session was to teach the students to accept and let go. Acceptance and letting things take their course are psychological adjustment strategies that emphasize adopting an open and non-confrontational attitude toward difficulties, challenges, or uncomfortable feelings in life. Instead of trying to control or change these situations, or having excessive emotional reactions, it is about maintaining a peaceful state of mind and engaging in adaptive behaviors to promote inner harmony and psychological health. Practice mindfulness meditation first and then talk to the participants about accepting and letting go and getting along with difficulties. While working on MT’s fourth metaphor, the shark metaphor, participants first imagined themselves as sharks swimming in the ocean surrounded by many other fish, some larger and some smaller than themselves. First, the participant encountered a fish much larger than him or herself. Then, the fish were chased and allowed to escape. Later, they were confronted with a group of fish that were smaller than themselves, all of which were their dinner, and the participants tried their best to chase them. The participants were encouraged to experience their feelings in the two conflicting situations. Finally, the participants were asked to imagine that they were unique sharks and that meeting big and small fish was a normal part of life, but it did not affect how they were most likely to behave. This metaphor is related to feelings of inferiority, and the purpose of using it was to help individuals overcome their sense of inferiority, face various difficulties in life with courage, and respond to them appropriately.

The main goals of the sixth session were to understand the mental framework, learn to distinguish between thoughts and facts and identify unhelpful thoughts. The mental framework refers to the psychological structures people use to understand and interpret experiences that affect information processing, emotional responses, and action choices. Learning to distinguish between thoughts and facts is one of the keys to mental framework abilities. It requires participants to identify objective facts—events or data that exist independently of personal interpretations and subjective thoughts—individual perspectives, interpretations, and beliefs about these facts. This ability to differentiate helps participants assess situations more objectively, reduce cognitive biases, make more reasonable decisions, and enhance their adaptability in facing challenges. After studying and body scanning, the stone metaphor exercise was performed on MT. The goal was to consolidate and maintain the nonjudgmental attitude learned in the previous exercises. The metaphorical exercise asked the participants to imagine themselves as boulders. Sitting on top of the mountain by the sea, one has a wide view, watching the clouds rolling around, flowers blooming and falling, the ebb and flow of the sea, and the changes in the wind and clouds. However, one is still very stable. Lead participants were to feel the power of stability in this metaphorical story. The purpose of this metaphor is primarily to encourage participants to reflect upon and enhance their own worldviews and perspectives on life.

The seventh intervention helped participants understand the relationship between their behavior and emotions. The participants were instructed to turn kindness into action, record their own activities, identify whether the activity was friendly or depleting, list controlling and pleasurable activities, and use pleasurable and mastery activities to combat negative emotions. Simultaneously, participants were guided to integrate the five metaphors into MT.

The purpose of the last intervention was to provide a summative insight. After a period of mindfulness meditation, the participants were guided to share the changes in their mental processes during treatment. A second evaluation was conducted after the event. Three months after the last intervention, all the participants underwent a final follow-up assessment. Finally, the data were collated and entered into SPSS (version 25.0) for statistical analysis.

### 2.5 Results

The intervention and control groups were assessed thrice using descriptive statistics and a difference test (**Table 3**). The results showed no significant differences between the intervention and control groups, indicating that the two groups were homogenous and could be used as the experimental and control groups, respectively. The scores of the intervention group were significantly lower than those of the control group (T = -4.51, *p* < 0.001). The difference between the two groups was significant: the scores in the intervention group were significantly lower than those in the control group.

**Table 3:** Descriptive statistics of the total mean score and the mean score of each dimension of PSMU.

Variable	Experimental group (*n* = 30)			Control group (*n* = 30)		
	Pre-test	Post-test	Follow-up	Pre-test	Post-test	Follow-up
PSMU-total score	4.00 ± 0.36	3.16 ± 0.56	3.26 ± 0.48	3.89 ± 0.70	3.88 ± 0.76	3.91 ± 0.62
Increased viscosity	4.23 ± 0.51	3.39 ± 0.75	3.38 ± 0.66	4.13 ± 0.69	4.05 ± 0.80	4.13 ± 0.78
Physiological damage	3.77 ± 0.59	3.05 ± 0.81	3.13 ± 0.67	3.77 ± 0.86	3.83 ± 0.85	3.85 ± 0.58
Omission anxiety	4.06 ± 0.42	3.15 ± 0.71	3.42 ± 0.77	3.73 ± 0.94	3.79 ± 1.03	3.83 ± 0.97
Cognitive failure	4.01 ± 0.49	3.23 ± 0.71	3.39 ± 0.66	3.78 ± 0.83	3.82 ± 0.83	3.99 ± 0.67
Guilt	3.87 ± 0.96	2.73 ± 0.88	2.96 ± 0.85	4.13 ± 0.85	3.87 ± 1.06	3.73 ± 1.07

To further test the effect of the intervention, a 2 (intervention and control groups) × 3 (pre-test, post-test, and follow-up test) multivariate ANOVA was performed on both groups. The results are summarized in **Table 4**.

**Table 4:** Results of ANOVA.

	Variable	*F*	*P*	*η_p_^2^*
Time	PSMU-total score	32.444	< 0.001	0.699
	Increased viscosity	22.621	< 0.001	0.618
	Physiological damage	10.163	< 0.001	0.421
	Omission anxiety	17.581	< 0.001	0.557
	Cognitive failure	22.173	< 0.001	0.613
	guilt	20.339	< 0.001	0.592
Groups	PSMU-total score	10.469	0.003	0.265
	Increased viscosity	11.467	0.002	0.283
	Physiological damage	7.623	0.010	0.208
	Omission anxiety	1.426	0.242	0.047
	Cognitive failure	4.861	0.036	0.144
	guilt	22.939	< 0.001	0.442
Time × Groups	PSMU-total score	23.614	< 0.001	0.628
	Increased viscosity	13.382	< 0.001	0.489
	Physiological damage	11.727	< 0.001	0.456
	Omission anxiety	17.284	< 0.001	0.552
	Cognitive failure	19.350	< 0.001	0.580
	guilt	8.859	0.001	0.388

First, the results showed that the spherical hypothesis was valid (*p* < 0.05), and the Greenhouse–Geisser method was used to correct the results. The time main effect, group main effect, and interaction were significant, indicating that social media use of the problem participants significantly improved the intervention process based on mindfulness cognitive therapy combined with MT. Further simple effect analysis found that the difference between the intervention group and the control group in the pre-test score was not significant [F(1,58) = 1.29, *p* = 0.261, ηp2 = 0.022]; the intervention group and the control group had a significant difference in the post-test score [F(1,58) = 20.38, *p* < 0.00 01, ηp2 = 0.260], and the post-test score of the intervention group is lower than that of the control group; the tracking score of the intervention group and the control group is significantly different [F(1,58) = 19.654, *p* < 0.0001, ηp2 = 0.253], and the tracking score of the intervention group is significantly lower than that of the control group. The score difference between the pre-test, post-test, and follow-up test in the control group is not significant [F(2,57) = 0.936, *p* = 0.398, ηp2 = 0.032]; the difference between the pre-test, post-test and follow-up score of the intervention group is significant [F(2,57) = 15.668, *p* < 0.000 1, ηp2 = 0.36]; the specific performance is that the post-test score of the intervention group is significantly lower than the pre-test and the tracking score (PSMU was reduced by 29.5%), and the tracking test score is also significantly lower than the pre-test score (reduced by 14.5%). The participants also self-reported their social media usage time before and after the intervention. The experimental group’s average daily social media usage time decreased from over four h to no more than two h, whereas there was no significant change in the usage time for the control group, indicating that mindfulness cognitive therapy combined with MT can effectively improve PSMU and that this effect is persistent.

## 3 Discussion

This study showed that MBCT combined with MT significantly affected PSMU. This suggests that, in addition to traditional intervention programs that rely on behavior control (14), cognitive change-based intervention approaches can also positively affect PSMU. This is because the compensatory Internet use theory (10), I-PACE model (11), uses and gratifications theory (12), and the cognitive-behavioral model (13) all highlight the causes of PSMU. Some of the most critical points are an individual’s psychological needs, intrinsic motivation, and irrational cognition. Although interventions at the behavioral level can achieve results in a short period, it is necessary to explore the individual’s psychological needs and intrinsic motivation to change irrational cognition. Learning to embrace and live in the present can fundamentally improve individuals’ PSMU behavior.

The MBCT was the main intervention protocol used in this study and was the main factor. There were several reasons for this observation. First, MBCT can help individuals develop a non-critical observer perspective, which is important for mitigating comparisons and envy caused by social media. On social media, individuals display only the best aspects of their lives, which can cause them to feel dissatisfied or devalued. Mindfulness reduces unnecessary self-deprecation and envy by fostering a nonjudgmental attitude that enables individuals to process information on social media more objectively and rationally (19). Second, MBCT can improve an individual’s ability to perceive and regulate their own emotions and behaviors by improving their level of mindfulness, which means that individuals can effectively identify and manage negative emotions, such as guilt and anxiety, associated with social media use. Simultaneously, increased self-regulation can help individuals develop healthy social media habits (34). Third, mindfulness training covers the full range of life experiences and helps individuals reduce the “fear of loss” associated with social media use. By fostering acceptance of current experiences, one can focus more on one’s own life and experiences than on the dynamics of others (19). Fourth, MBCT can also help individuals identify and adjust to materialism by promoting a focus on intrinsic values and personal growth, helping them build healthier and more balanced value systems, thus reducing dissatisfaction and anxiety caused by social media (19).

The reasons why MT can alleviate PSMU to some extent may include the following points: first, emotional resonance and empathy. MT uses stories and metaphors to create emotional resonance, allowing people to discuss their feelings and behaviors without directly touching on sensitive topics. This indirectness may make it easier for those who feel uncomfortable or defensive about discussing their social media use. Second, it provides self-reflection and insights. By engaging in metaphorical stories, individuals develop these qualities. They may see themselves reflected in the characters or situations in the stories, which can lead to an in-depth consideration of their actions and emotions. For example, the five metaphorical stories used by this researcher involve personal goals, coping with setbacks, parent-child relationships, and self-evaluation, all of which can provoke insights among participants. Third, there were behavioral changes. MT can also guide individuals in exploring and practicing new behavioral patterns. By simulating these new behaviors within metaphorical stories, individuals can learn how to apply these changes to real life (32).

The main reason for combining MBCT and MT in this study was that both psychotherapy methods can act on both cognitive and behavioral aspects (32). Simultaneously, the two methods used in the process complemented each other. In MBCT, individuals can discover their own irrational cognition and learn to face facts directly in the process of experiencing and paying attention to the present; through five metaphorical stories with conflicting characteristics, the participants were able to transform themselves into a state of contradiction and peace, and in this process, strengthen their attitude toward living in the present without judgment (32). Therefore, MT enhances the interventional effects of MBCT to a certain extent.

This study investigated the potential of ameliorating PSMU by integrating MBCT and MT. Preliminary findings indicated that this integrated approach positively influenced the frequency of social media engagement and bolstered individuals’ self-regulatory capacities. However, this study had several limitations. First, the sample size and diversity were constrained, with the study participants predominantly from a specific age group and cultural context, thereby compromising the generalizability and representativeness of the findings. Second, the long-term efficacy of the therapy remains undetermined as the study was based on short-term observational data and did not assess the duration of therapeutic benefits. Finally, there is a paucity of understanding of individual differences in social media usage patterns and psychological underpinnings, which could impede the tailored application of therapy. To enhance the external validity of this study, future studies should recruit a more diverse cohort across age, culture, and psychological backgrounds. Longitudinal studies should also be conducted to evaluate the durability of therapeutic effects and the potential for relapse. Moreover, by comprehensively examining individual differences, tailored treatment plans can be formulated to augment therapeutic efficacy. As previously highlighted, PSMU and Internet addiction may also share similar psychological mechanisms, albeit to varying degrees (3). Hence, theoretically, the combination of MBCT and MT could be effective in treating Internet addiction. However, this hypothesis requires further empirical validation.

## 4 Conclusion

This study explores the effectiveness of improving PSMU by integrating MBCT and MT. The results indicate that this innovative treatment approach significantly reduces participants’ frequency of social media use, enhances their self-regulation capabilities, and decreases anxiety and depressive symptoms associated with social media use. Through mindfulness exercises, participants learned to recognize better and accept their emotional responses, whereas MT helped them understand and express the psychological motivations behind social media use in a more symbolic and creative manner. The combination of these therapies not only provides a novel psychological intervention but also reveals the synergistic effects of mindfulness and metaphors in altering harmful behavioral patterns. Although this study has some limitations, such as a small sample size and lack of long-term follow-up data, these preliminary results lay the foundation for future larger-scale and more in-depth research and offer valuable insights for clinical practice in reducing PSMU. Overall, this study suggests that the combination of MBCT and MT is a promising therapeutic pathway with the potential to help individuals establish healthier social media usage habits and improve their psychological well-being.

## Data Availability

The original contributions presented in the study are included in the article/supplementary material. Further inquiries can be directed to the corresponding author.

## References

[1] Mieczkowski H Lee AY Hancock JT. Priming effects of social media use scales on well-being outcomes: the influence of intensity and addiction scales on self-reported depression. Soc Media+ Soc. (2020) 6:2056305120961784. doi: 10.1177/2056305120961784

[2] Gosling SD Augustine AA Vazire S Holtzman N Gaddis S. Manifestations of personality in Online Social Networks: self-reported Facebook-related behaviors and observable profile information. Cyberpsychology, Behavior, and Social Networking, Vol. 14. (2011). pp. 483–8. pp. 483–8. 10.1089/cyber.2010.0087 PMC3180765 21254929

[3] Jiang YZ Bai XL. Relationship between adolescent narcissistic personality and the use of problematic social networks: Analysis of chain intermediation. Special Educ China. (2020) 01):90–6. doi: 10.3969/j.issn.1007-3728.2020.01.015

[4] Sun GQ Yu Y Luo ZL Li Y Zhao HY. Research on mobile phone Internet addiction and Internet use self-control in middle school students. Chin J Health Psychol. (2011) 9:1078–80. doi: 10.13342/j.cnki.cjhp.2011.09.028

[5] Hu W Jiang YH Wang Q Wang N. The relationship between short video social media dependence and college students’ sleep disorders: the intermediary role of social media use at night and gender differences. Chin J Clin Psychol. (2021) 01):46–50. doi: 10.16128/j.cnki.1005-3611.2021.01.009

[6] Pi LY Li X. The relationship between college students’ loneliness and problematic mobile social media use: variable-centered and individual-centered analysis. Chin J Health Psychol. (2023) 31:936–42. doi: 10.13342/j.cnki.cjhp.2023.06.027

[7] Song H Zmyslinski-Seelig A Kim J Drent A Victor A Omori K. Does Facebook make you lonely?: A meta analysis. Comput Hum Behav. (2014) 36:446–52. doi: 10.1016/j.chb.2014.04.011

[8] Stavropoulos V Alexandraki K Motti-Stefanidi F. Recognizing internet addiction: Prevalence and relationship to academic achievement in adolescents enrolled in urban and rural Greek high schools. J Adolescence. (2013) 36:565–76. doi: 10.1016/j.adolescence.2013.03.008 23608781

[9] Kross E Verduyn P Demiralp E Park J Lee DS Lin N. Facebook use predicts declines in subjective well-being in young adults. PLoS One. (2013) 8:e69841. doi: 10.1371/journal.pone.0069841 23967061 PMC3743827

[10] Kardefelt-Winther D. A conceptual and methodological critique of internet addiction research: Towards a model of compensatory internet use. Comput Hum Behav. (2014) 31:351–4. doi: 10.1016/j.chb.2013.10.059

[11] Brand M Wegmann E Stark R Müllere A Wölfling K Robbins TW. The Interaction of person-affect-cognition-execution (I-PACE) model foraddictive behaviors: Update, generalization to addictive behaviors beyond internet use disorders,and specification of the process character of addictive behaviors. Neurosci Biobehav Rev. (2019) 104:1–10. doi: 10.1016/j.neubiorev.2019.06.032 31247240

[12] Blumler J. The role of theory in uses and gratifications studies. Communication Res. (1979) 6:9–36. doi: 10.1177/009365027900600102

[13] Davis RA. A cognitive-behavioral model of pathological Internet use. Comput Hum Behav. (2001) 17:187–95. doi: 10.1016/S0747-5632(00)00041-8

[14] Stinson L Dallery J. Reducing problematic social media use via a package intervention. J Appl Behav Anal. (2023) 56:323–35. doi: 10.1002/jaba.v56.2 36782393

[15] Zhou X Rau P Yang C-L Zhou X. Cognitive behavioral therapy-based short-term abstinence intervention for problematic social media use: improved well-being and underlying mechanisms. Psychiatr Q. (2020) 92:761–79. (2021). doi: 10.1007/s11126-020-09852-0 32989636

[16] Chen Y Liu X Chiu DT Li Y Mi B Zhang Y. Problematic social media use and depressive outcomes among college students in China: observational and experimental findings. Int J Environ Res Public Health. (2022) 19:4937. doi: 10.3390/ijerph19094937 35564330 PMC9099455

[17] Lau MA McMain SF. Integrating Mindfulness Meditation With Cognitive and Behavioural Therapies: The challenge of combining acceptance- and change-based strategies. Can J Psychiatry. (2005) 50:863–70. doi: 10.1177/070674370505001310 16483122

[18] Kabat-Zinn J. Mindfulness-based interventions in context: Past, present, and future. Clinical Psychology: Science and Practice. (2003) 10:144–56. doi: 10.1093/clipsy.bpg016

[19] Kuyken W Watkins E Holden E White K Taylor R Byford S. How does mindfulness-based cognitive therapy work? Behav Res Ther. (2010) 48:1105–12. doi: 10.1016/j.brat.2010.08.003 20810101

[20] Liu C Chen H Liu CY Lin R Chiou WK. Effects of loving-kindness meditation on mindfulness, spirituality and subjective well-being of flight attendants. Cross-Cultural Design. Appl Health Learning Communication Creativity. (2020) 12193:151–65. doi: 10.1007/978-3-030-49913-6_13

[21] Liu C Chen H Zhang A Gong XG Wu K Liu CY. The effects of short video app-guided loving-kindness meditation on college students’ mindfulness, self-compassion, positive psychological capital, and suicide ideation. Psicologia: Re exão e Crítica. (2023) 36:32. doi: 10.1186/s41155-023-00276-w PMC10616025 37902928

[22] Chiou WK Hsu SE Liang YC Hong TH Lo LM Chen H. ISDT case study of we’ll app for postpartum depression women. In: Cross-Cultural Design. Applications in Health, Learning, Communication, and Creativity, vol. 12772. (2021). p. 119–37. Cham: Springer Nature Switzerland AG. doi: 10.1007/978-3-030-77077-8_10

[23] Liu C Chen H Liang YC Lin R Chiou WK. ISDT case study of loving kindness meditation for flight attendants. In: Cross-Cultural Design. Applications in Health, Learning, Communication, and Creativity, vol. 12772. (2021). p. 201–16. Cham: Springer Nature Switzerland AG. doi: 10.1007/978-3-030-77077-8_16

[24] Chen H Liu C Hsu SE Huang DH Liu CY Chiou WK. The effects of animation on the guessability of universal healthcare symbols for middle-aged and older adults. Hum Factors. (2021) 65:1740–58. doi: 10.1177/00187208211060900 34969321

[25] Chiou WK Liu C Chen H Hsu SE. Reliability and validity assessment of the Chinese version of flow ergonomics. Cross-Cultural Design. Interaction Design Across Cultures. (2022) 13311:330–41. doi: 10.1007/978-3-031-06038-0_24

[26] Jones A Hook M Podduturi P McKeen H Beitzell E Liss M. Mindfulness as a mediator in the relationship between social media engagement and depression in young adults. Pers Individ Dif. (2022) 185:111284. doi: 10.1016/j.paid.2021.111284

[27] Gómez L Molina ES Collazos CA de Pinho LG. Technological system based on serious games for teaching stress management to university students. IEEE Rev Iberoamericana Tecnologias del Aprendizaje. (2024) 19:248–57. doi: 10.1109/rita.2024.3465044

[28] Kopp RR. Metaphor therapy: Using clientgenerated metaphors in psychotherapy. London: Taylor & Francis Group (1995).

[29] Kopp RR Craw MJ. Metaphoric language, metaphoric cognition, and cognitive therapy. Psychotherapy. (1998) 35:306–11. doi: 10.1037/h0087795

[30] Stoddard JA Afari N. The big book of ACT metaphors: A practitioner’s guide to experiential exercises and metaphors in acceptance and commitment therapy. Oakland: New Harbinger Publications (2014).

[31] Komasi S Saeidi M Zakiei A Amiri MM Soltani B. Cognitive restructuring based on metaphor therapy to challenge the irrational beliefs of drug addicts undergoing buprenorphine treatment. Int J High Risk Behav Addict. (2016) 6:e31450. doi: 10.5812/ijhrba.31450

[32] Xiong WR. Return to the original state. Beijing: Beijing Normal University Press (2021).

[33] Jiang YZ. Adolescent problem mobile social media use evaluation questionnaire compiled. psychol Technol Appl. (2018) 10):613–21. doi: 10.16842/j.cnki.issn2095-5588.2018.10.004

[34] Liu JJ Duan W Xiao ZP Wu YR. The efficacy of patients with depression participating in online mindfulness cognitive therapy and the relationship between depressive symptoms and fear self. Chin J Health Psychol. (2024) 05):641–5. doi: 10.13342/j.cnki.cjhp.2024.05.001

